# Supplementary material for: 3D texture-based face recognition system using fine-tuned deep residual networks
Source: PeerJ Comput Sci. 2019 Dec 2;5:e236. doi: 10.7717/peerj-cs.236 (PMC7924501; doi:10.7717/peerj-cs.236)
Supplement: Supplemental Information 5 [file peerj-cs-05-236-s005.zip › Source files-part1-Only for checking-To PeerJ-examiner-Please Download this Zip∩╝îAll the source files in my Manuscript-3D textures based face recognition--Author-SIMING ZHENG/3---ALL Equation -- in my Manuscript --Source Document/4th-equation-files/3rd-LateX-Source Code.docx]

G(x,y)=\lbrack G_x(x,y)^2+G_y(x,y)^2\rbrack\;^{1/2}

$$G(x,y)=[G_{x}(x,y)^{2}+G_{y}(x,y)^{2}]{}^{1/2}$$
