# Supplementary material for: 3D texture-based face recognition system using fine-tuned deep residual networks
Source: PeerJ Comput Sci. 2019 Dec 2;5:e236. doi: 10.7717/peerj-cs.236 (PMC7924501; doi:10.7717/peerj-cs.236)
Supplement: Supplemental Information 5 [file peerj-cs-05-236-s005.zip › Source files-part1-Only for checking-To PeerJ-examiner-Please Download this Zip∩╝îAll the source files in my Manuscript-3D textures based face recognition--Author-SIMING ZHENG/3---ALL Equation -- in my Manuscript --Source Document/7th-equation-files/5th-LateX Source Code.docx]

h'=\lbrack\frac{h-f+s}s\rbrack,\;\;\;\;w'=\lbrack\frac{w-f+s}s\rbrack.\;

$$h'=[\frac{h-f+s}{s}], w'=[\frac{w-f+s}{s}].$$
