# Supplementary material for: 3D texture-based face recognition system using fine-tuned deep residual networks
Source: PeerJ Comput Sci. 2019 Dec 2;5:e236. doi: 10.7717/peerj-cs.236 (PMC7924501; doi:10.7717/peerj-cs.236)
Supplement: Supplemental Information 5 [file peerj-cs-05-236-s005.zip › Source files-part1-Only for checking-To PeerJ-examiner-Please Download this Zip∩╝îAll the source files in my Manuscript-3D textures based face recognition--Author-SIMING ZHENG/3---ALL Equation -- in my Manuscript --Source Document/1st-equation-files/1st-equation.pdf]

$$I(x,y) = I(x,y)^{gamma}$$
