# Supplementary figures and images for: 3D texture-based face recognition system using fine-tuned deep residual networks
Source: PeerJ Comput Sci. 2019 Dec 2;5:e236. doi: 10.7717/peerj-cs.236 (PMC7924501; doi:10.7717/peerj-cs.236)

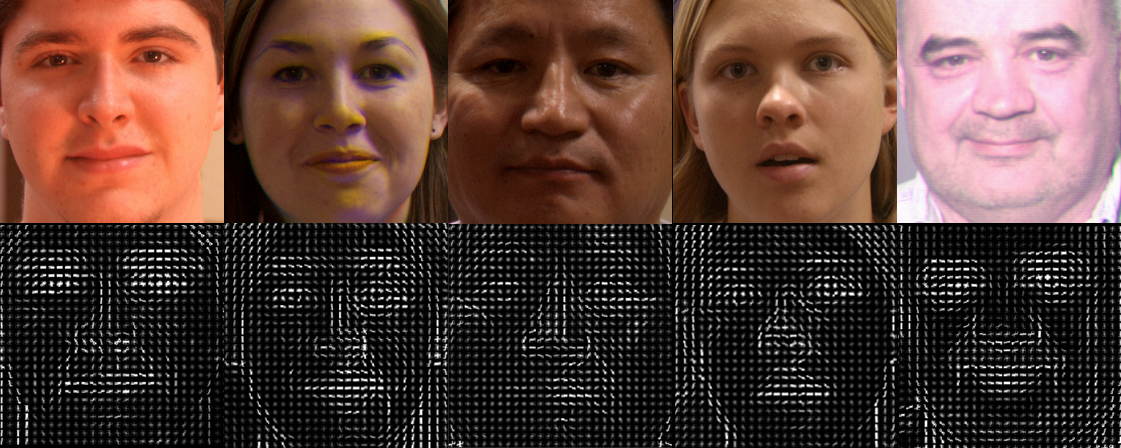

Supplement: Supplemental Information 5 [file peerj-cs-05-236-s005.zip › Source files-part1-Only for checking-To PeerJ-examiner-Please Download this Zip∩╝îAll the source files in my Manuscript-3D textures based face recognition--Author-SIMING ZHENG/2---68-key-point--Source Document/5persons--HOG.png]

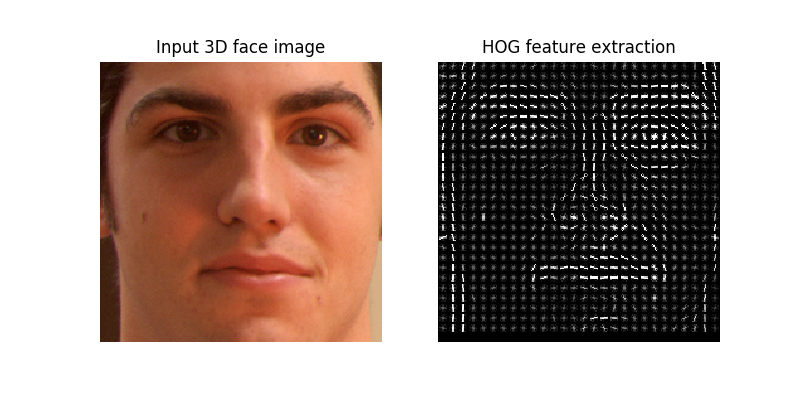

Supplement: Supplemental Information 5 [file peerj-cs-05-236-s005.zip › Source files-part1-Only for checking-To PeerJ-examiner-Please Download this Zip∩╝îAll the source files in my Manuscript-3D textures based face recognition--Author-SIMING ZHENG/2---68-key-point--Source Document/02-feils/test.png]

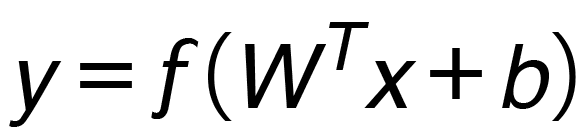

Supplement: Supplemental Information 5 [file peerj-cs-05-236-s005.zip › Source files-part1-Only for checking-To PeerJ-examiner-Please Download this Zip∩╝îAll the source files in my Manuscript-3D textures based face recognition--Author-SIMING ZHENG/3---ALL Equation -- in my Manuscript --Source Document/8th-equation-files/6th-equation.tif]

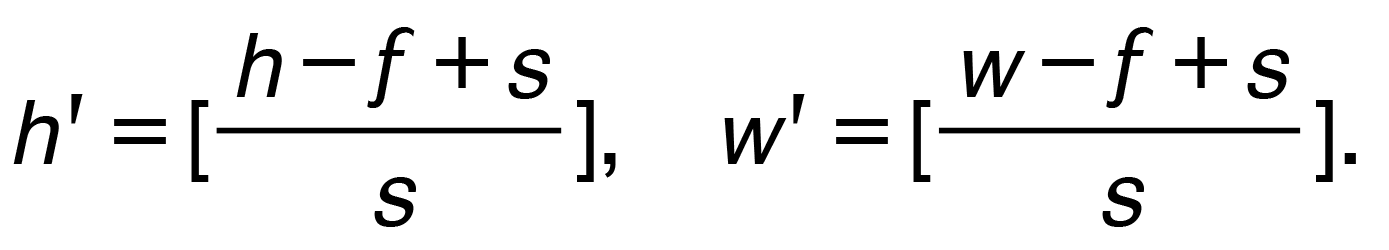

Supplement: Supplemental Information 5 [file peerj-cs-05-236-s005.zip › Source files-part1-Only for checking-To PeerJ-examiner-Please Download this Zip∩╝îAll the source files in my Manuscript-3D textures based face recognition--Author-SIMING ZHENG/3---ALL Equation -- in my Manuscript --Source Document/7th-equation-files/5th-equation.tif]

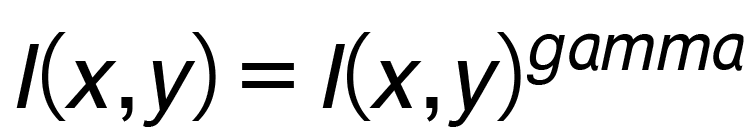

Supplement: Supplemental Information 5 [file peerj-cs-05-236-s005.zip › Source files-part1-Only for checking-To PeerJ-examiner-Please Download this Zip∩╝îAll the source files in my Manuscript-3D textures based face recognition--Author-SIMING ZHENG/3---ALL Equation -- in my Manuscript --Source Document/1st-equation-files/1st-equation.tif]

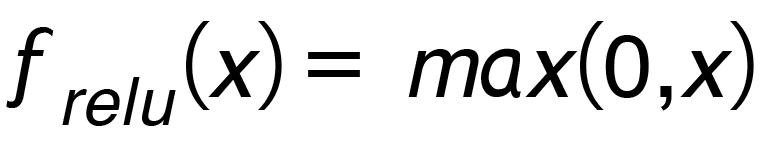

Supplement: Supplemental Information 5 [file peerj-cs-05-236-s005.zip › Source files-part1-Only for checking-To PeerJ-examiner-Please Download this Zip∩╝îAll the source files in my Manuscript-3D textures based face recognition--Author-SIMING ZHENG/3---ALL Equation -- in my Manuscript --Source Document/6th-equation-files/4th-equation.tif]

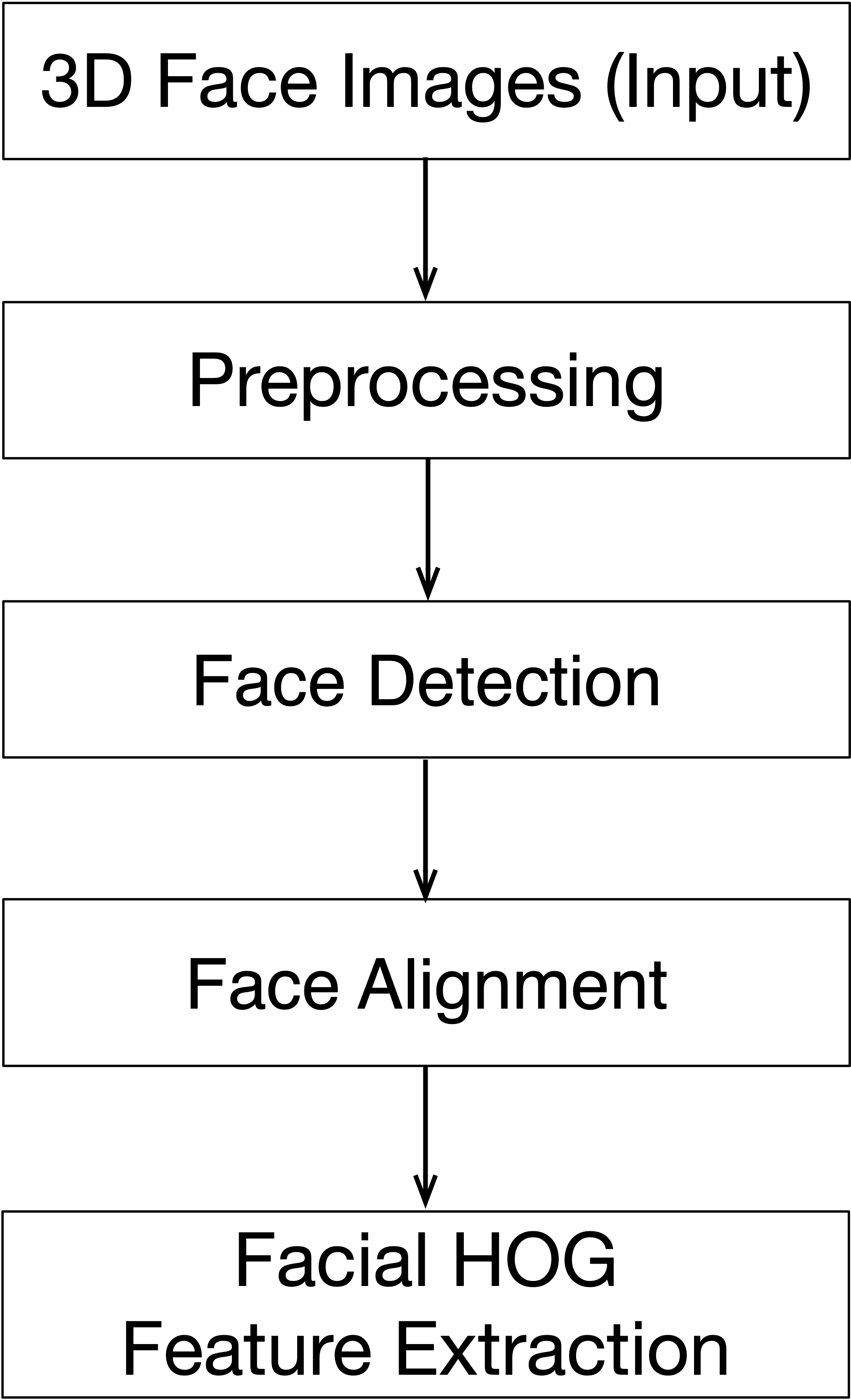

Supplement: Supplemental Information 5 [file peerj-cs-05-236-s005.zip › Source files-part1-Only for checking-To PeerJ-examiner-Please Download this Zip∩╝îAll the source files in my Manuscript-3D textures based face recognition--Author-SIMING ZHENG/1---3D face preprocessing -- Source Document/Preprocessing Approach Description.tiff]

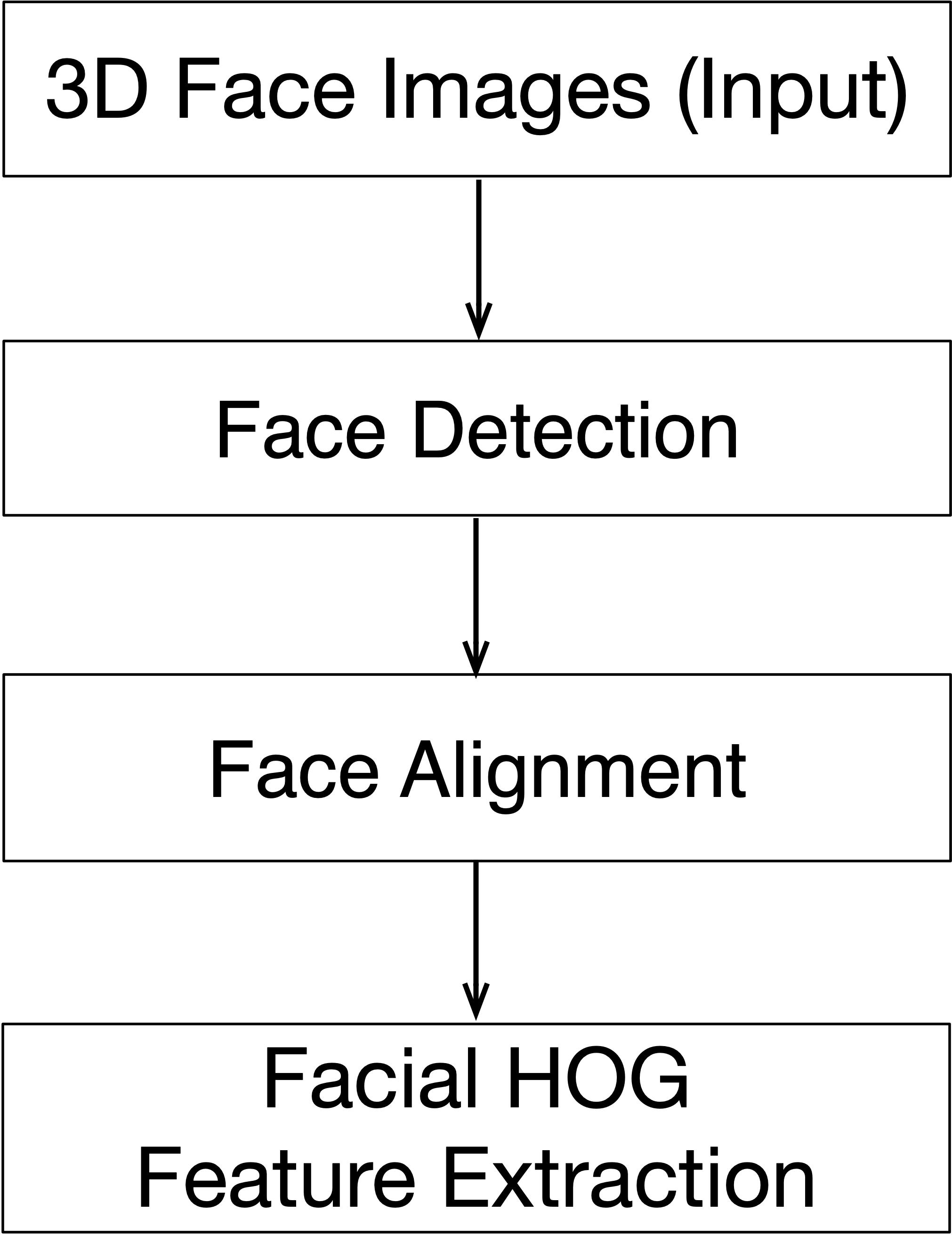

Supplement: Supplemental Information 5 [file peerj-cs-05-236-s005.zip › Source files-part1-Only for checking-To PeerJ-examiner-Please Download this Zip∩╝îAll the source files in my Manuscript-3D textures based face recognition--Author-SIMING ZHENG/1---3D face preprocessing -- Source Document/Preprocessing Approach Description-updated-2.png]

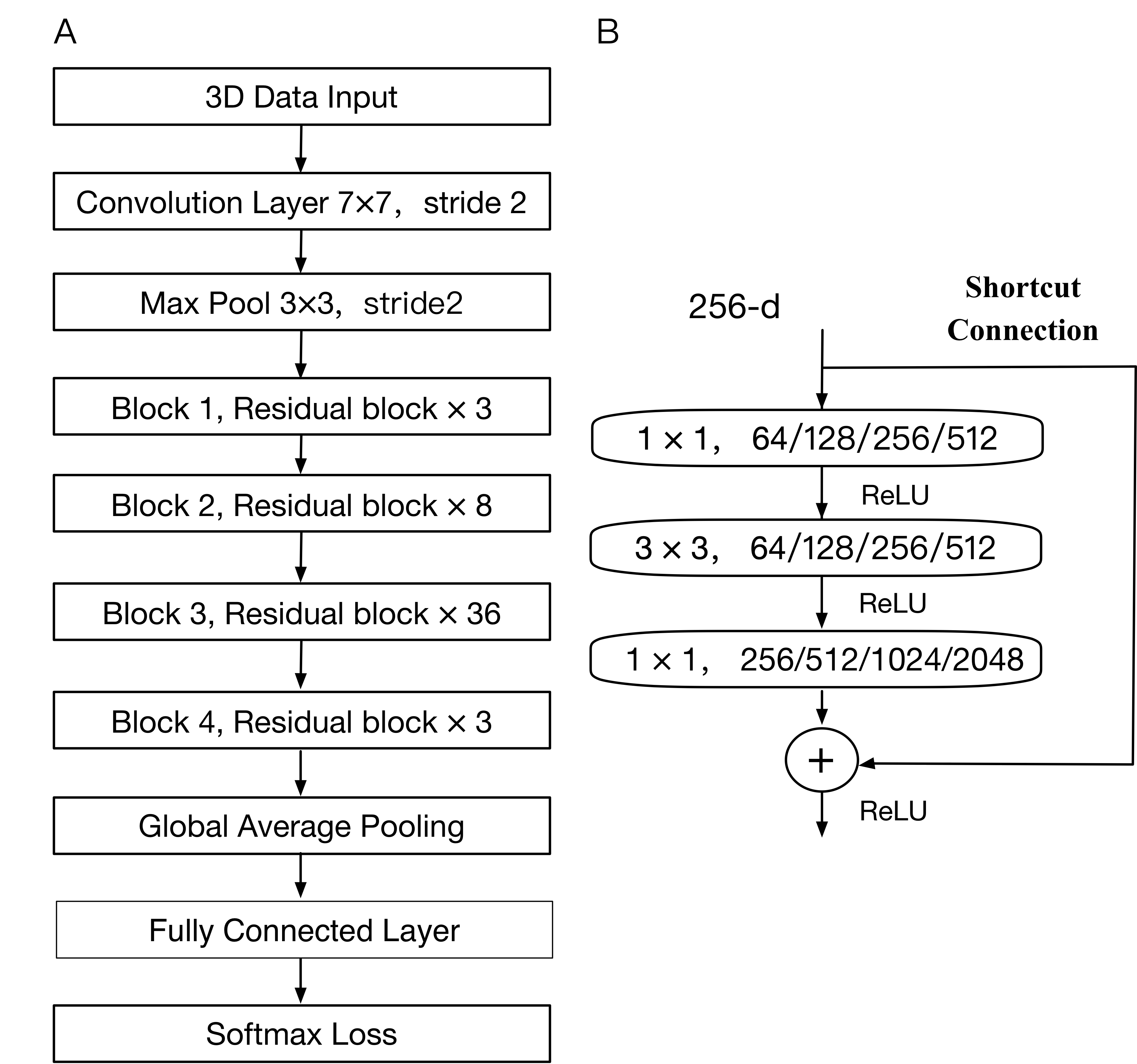

Supplement: Supplemental Information 6 [file peerj-cs-05-236-s006.zip › Source files-part2-Only for checking-To PeerJ-examiner-Please Download this Zip∩╝îAll the source files in my Manuscript-3D textures based face recognition--Author-SIMING ZHENG/4---ResNet-152layers-structure-figure--Source Document/resnet-152--.png]

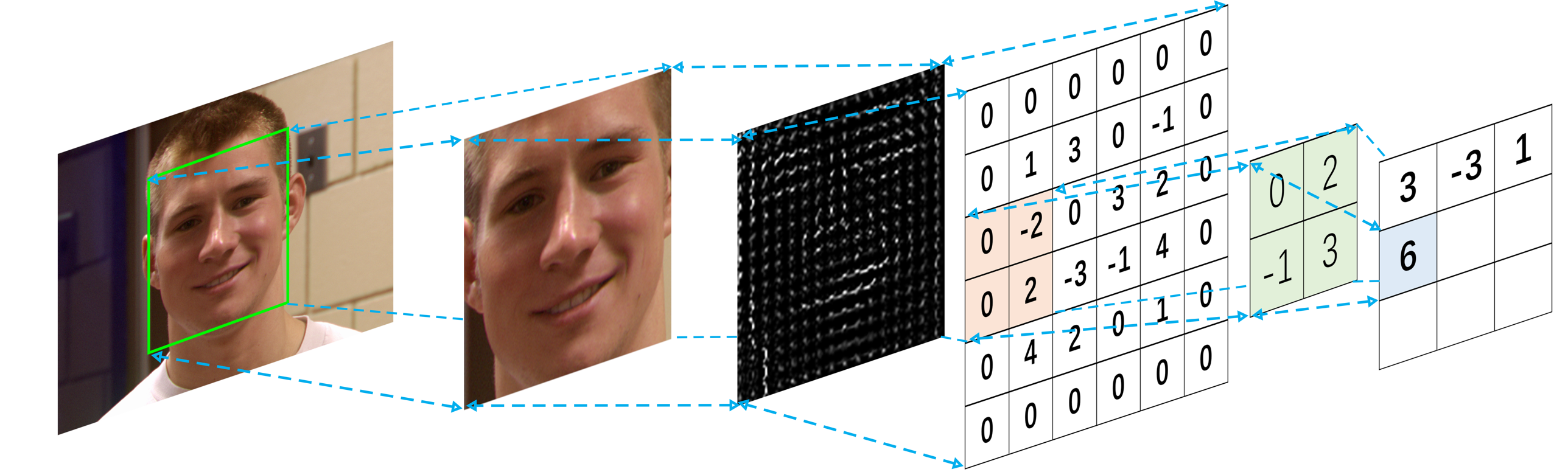

Supplement: Supplemental Information 6 [file peerj-cs-05-236-s006.zip › Source files-part2-Only for checking-To PeerJ-examiner-Please Download this Zip∩╝îAll the source files in my Manuscript-3D textures based face recognition--Author-SIMING ZHENG/4---Convolution operation figure--Source Document/juzhen-juanji-new.jpg]

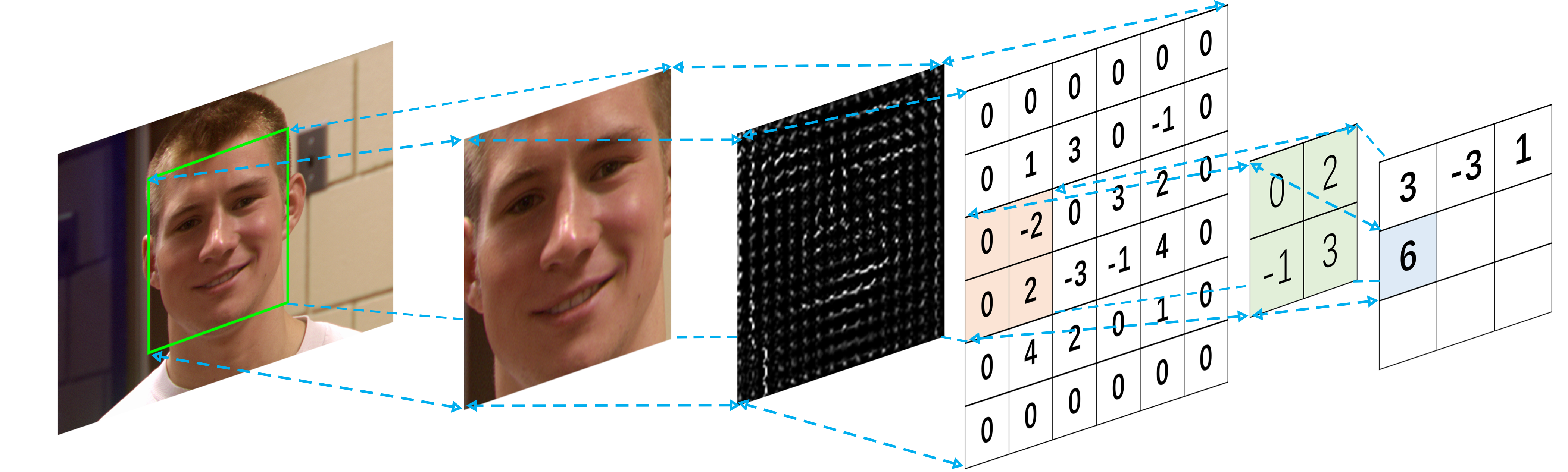

Supplement: Supplemental Information 6 [file peerj-cs-05-236-s006.zip › Source files-part2-Only for checking-To PeerJ-examiner-Please Download this Zip∩╝îAll the source files in my Manuscript-3D textures based face recognition--Author-SIMING ZHENG/4---Convolution operation figure--Source Document/juzhen-juanji--last one.png]

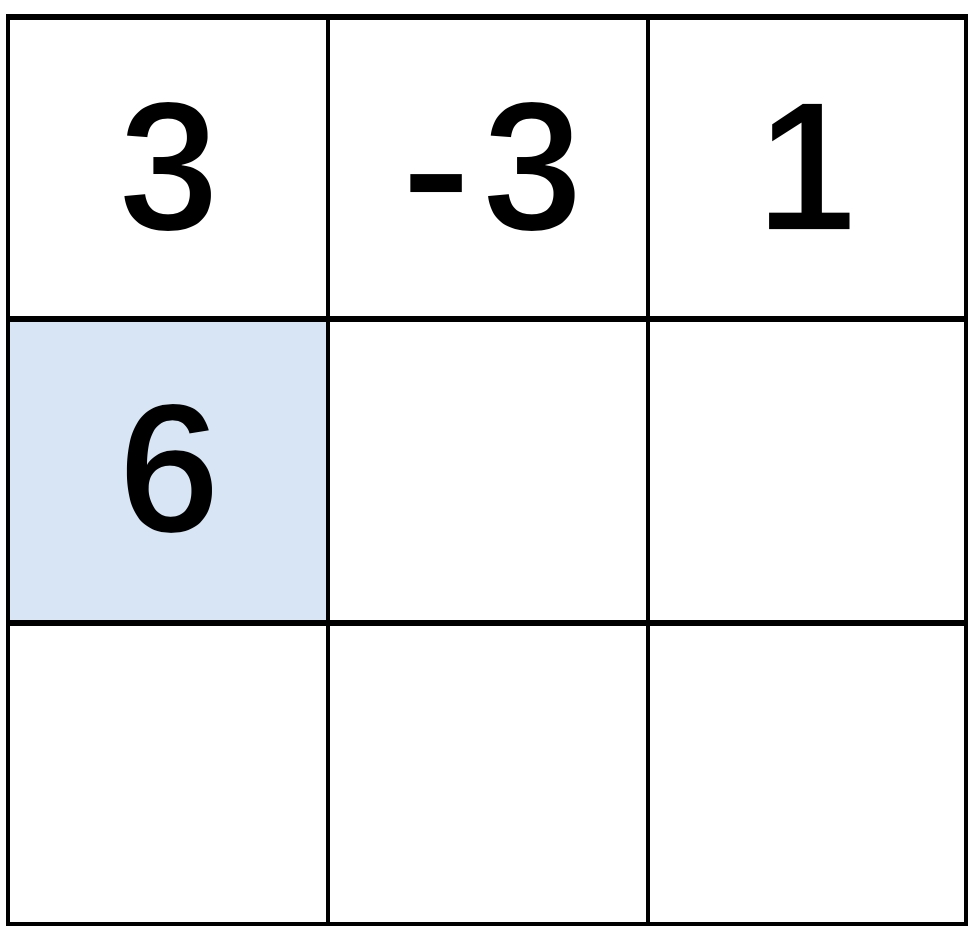

Supplement: Supplemental Information 6 [file peerj-cs-05-236-s006.zip › Source files-part2-Only for checking-To PeerJ-examiner-Please Download this Zip∩╝îAll the source files in my Manuscript-3D textures based face recognition--Author-SIMING ZHENG/4---Convolution operation figure--Source Document/juzhen3.png]

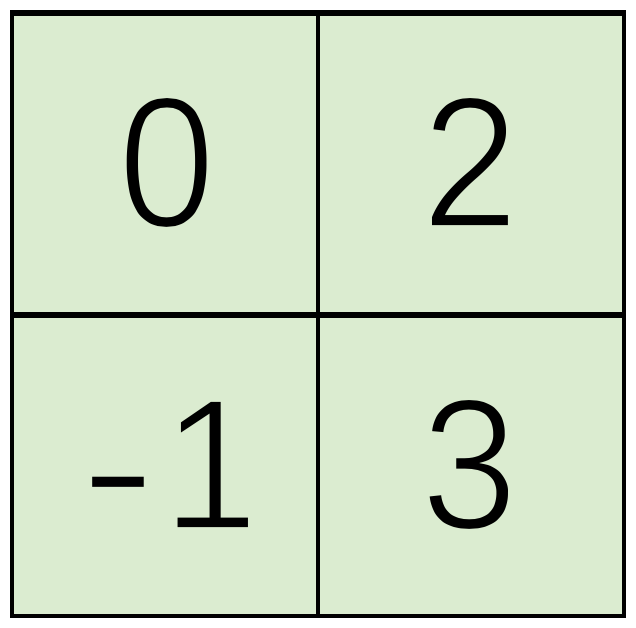

Supplement: Supplemental Information 6 [file peerj-cs-05-236-s006.zip › Source files-part2-Only for checking-To PeerJ-examiner-Please Download this Zip∩╝îAll the source files in my Manuscript-3D textures based face recognition--Author-SIMING ZHENG/4---Convolution operation figure--Source Document/juzhen2.png]

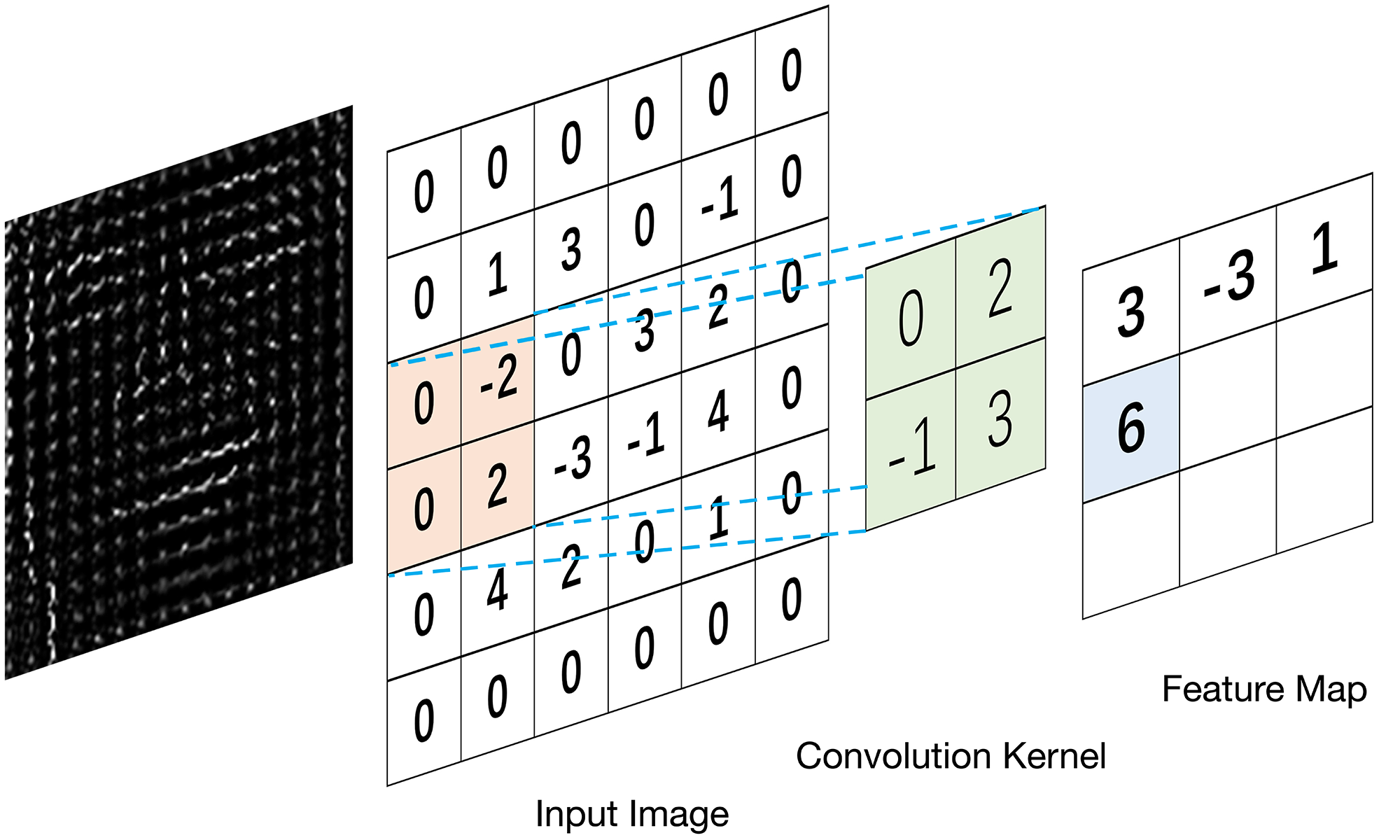

Supplement: Supplemental Information 6 [file peerj-cs-05-236-s006.zip › Source files-part2-Only for checking-To PeerJ-examiner-Please Download this Zip∩╝îAll the source files in my Manuscript-3D textures based face recognition--Author-SIMING ZHENG/4---Convolution operation figure--Source Document/convolution-operation.tiff]

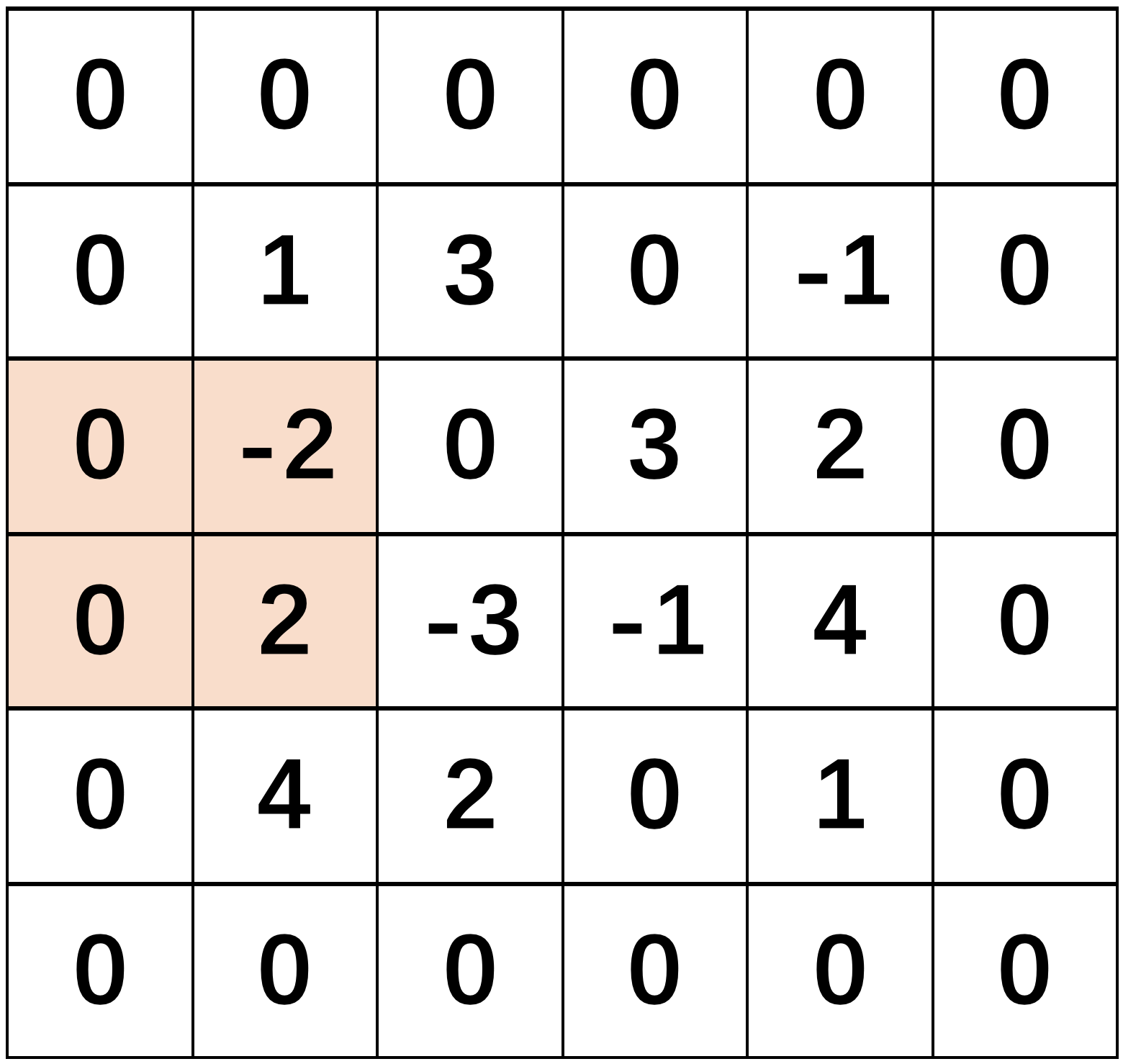

Supplement: Supplemental Information 6 [file peerj-cs-05-236-s006.zip › Source files-part2-Only for checking-To PeerJ-examiner-Please Download this Zip∩╝îAll the source files in my Manuscript-3D textures based face recognition--Author-SIMING ZHENG/4---Convolution operation figure--Source Document/juzhen1.png]

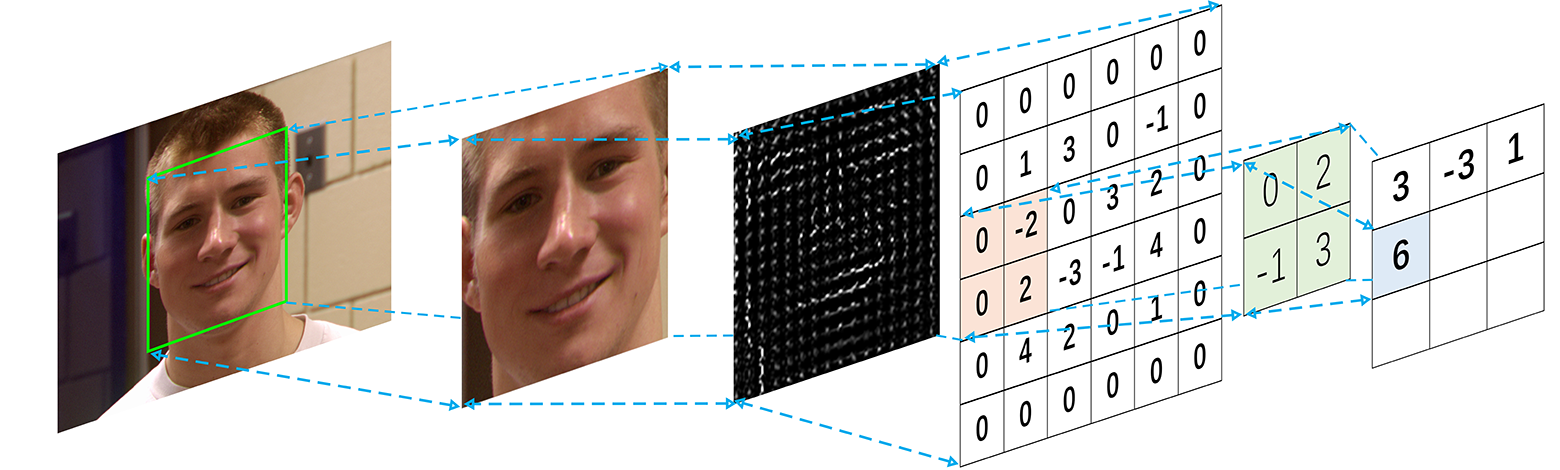

Supplement: Supplemental Information 6 [file peerj-cs-05-236-s006.zip › Source files-part2-Only for checking-To PeerJ-examiner-Please Download this Zip∩╝îAll the source files in my Manuscript-3D textures based face recognition--Author-SIMING ZHENG/4---Convolution operation figure--Source Document/juzhen-juanji-new-size.tif]

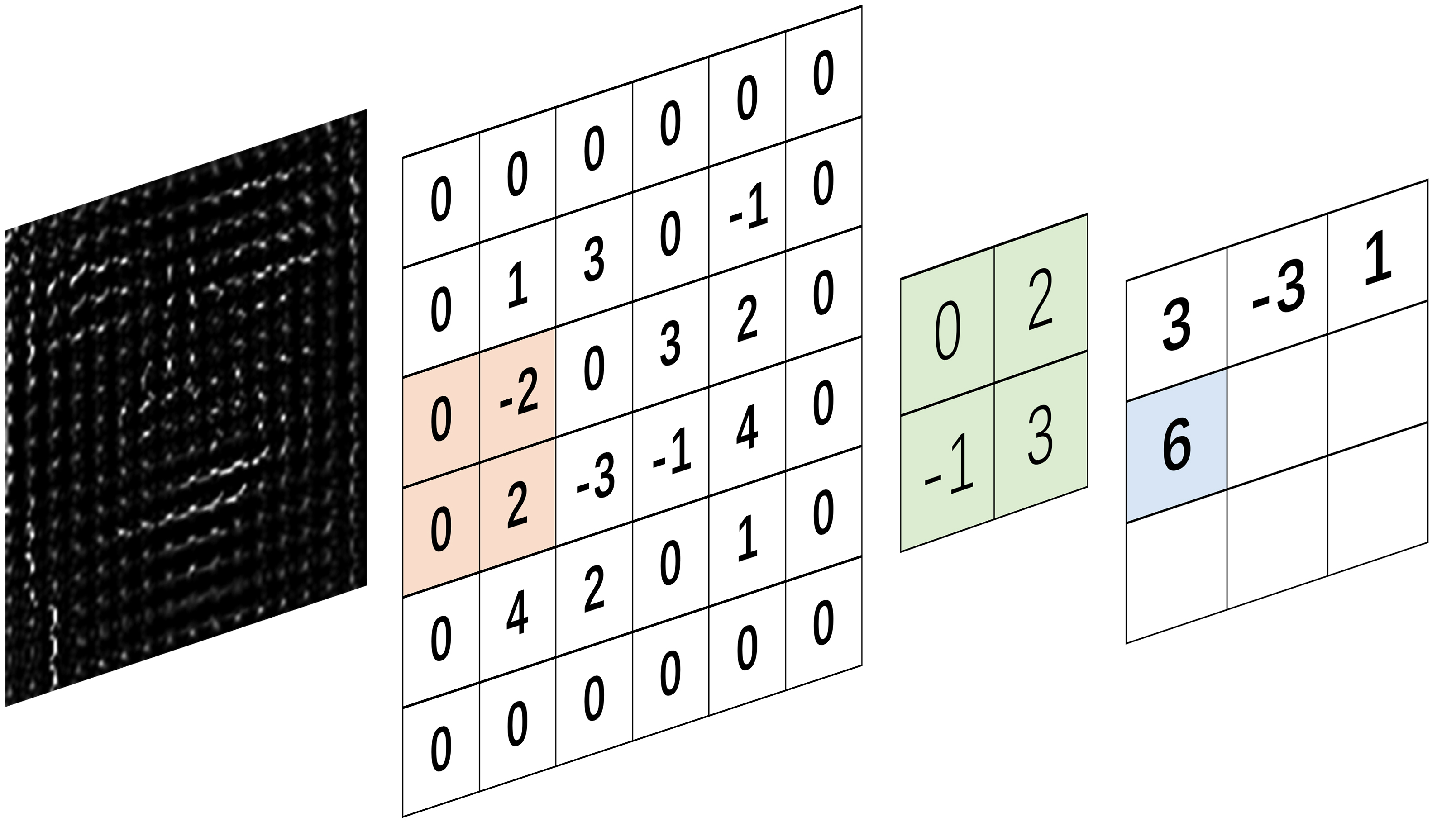

Supplement: Supplemental Information 6 [file peerj-cs-05-236-s006.zip › Source files-part2-Only for checking-To PeerJ-examiner-Please Download this Zip∩╝îAll the source files in my Manuscript-3D textures based face recognition--Author-SIMING ZHENG/4---Convolution operation figure--Source Document/juzhen-juanji.jpg]

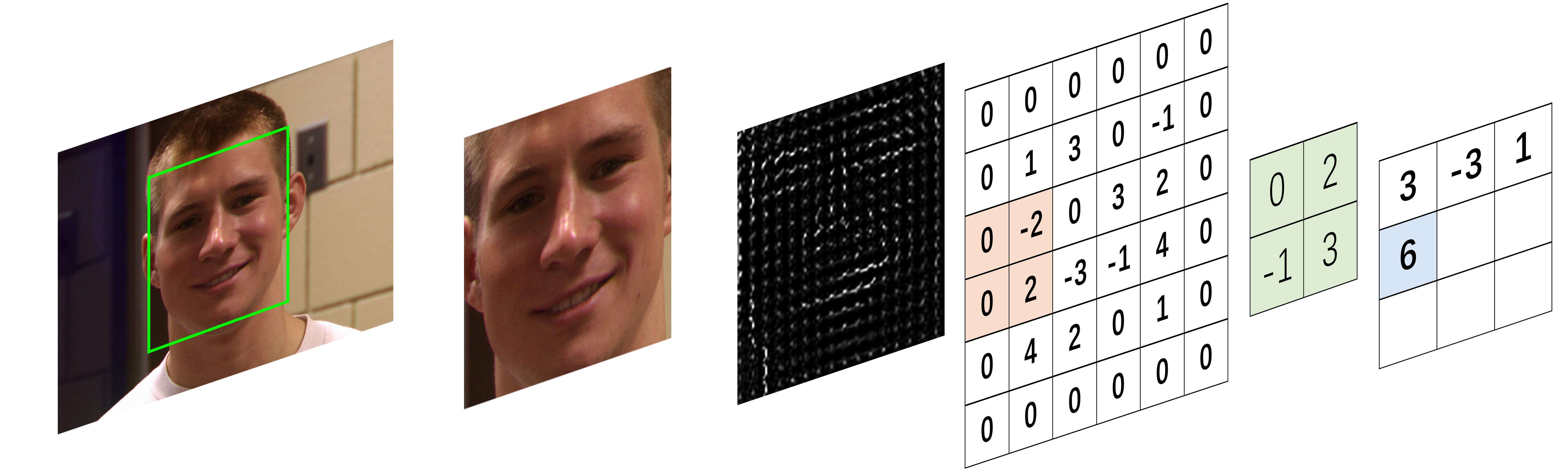

Supplement: Supplemental Information 6 [file peerj-cs-05-236-s006.zip › Source files-part2-Only for checking-To PeerJ-examiner-Please Download this Zip∩╝îAll the source files in my Manuscript-3D textures based face recognition--Author-SIMING ZHENG/4---Convolution operation figure--Source Document/juzhen-juanji2.jpg]

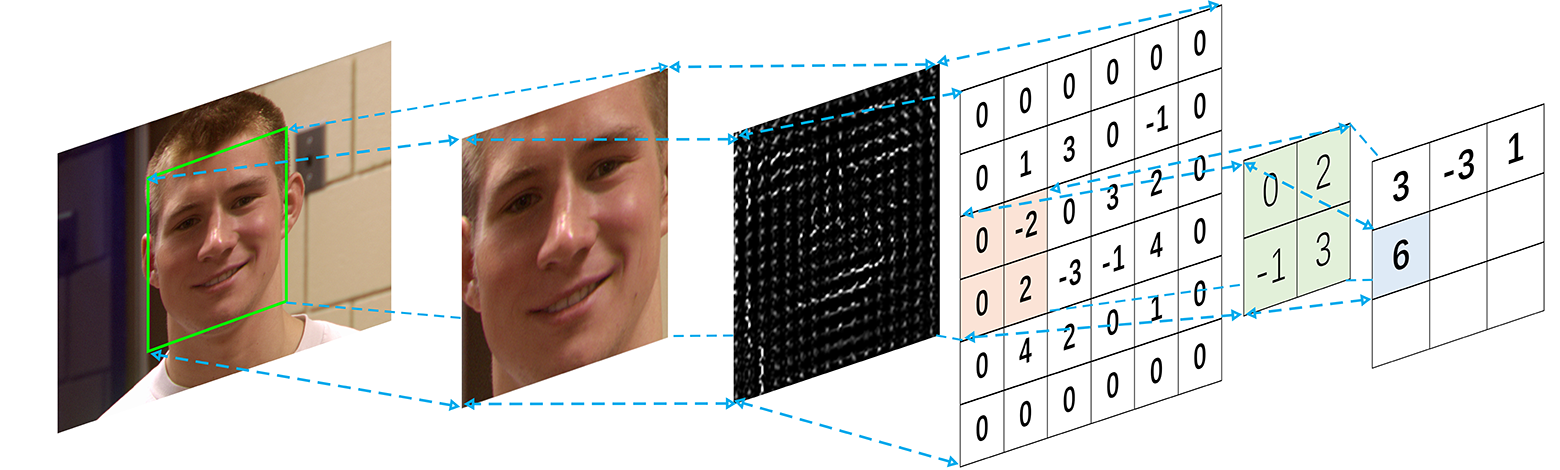

Supplement: Supplemental Information 6 [file peerj-cs-05-236-s006.zip › Source files-part2-Only for checking-To PeerJ-examiner-Please Download this Zip∩╝îAll the source files in my Manuscript-3D textures based face recognition--Author-SIMING ZHENG/4---Convolution operation figure--Source Document/juzhen-juanji-new-size.png]

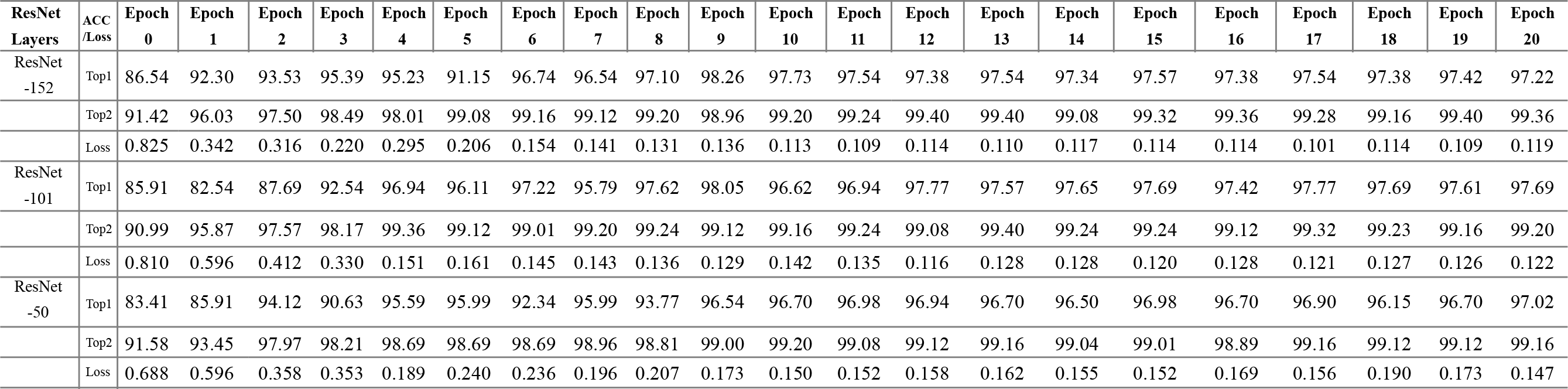

Supplement: Supplemental Information 7 [file peerj-cs-05-236-s007.zip › Source files-part3-Only for checking-To PeerJ-examiner-Please Download this Zip∩╝îAll the source files in my Manuscript-3D textures based face recognition--Author-SIMING ZHENG/6---Table-data-files---Source Document--50-101-152/result-table-A.tif]

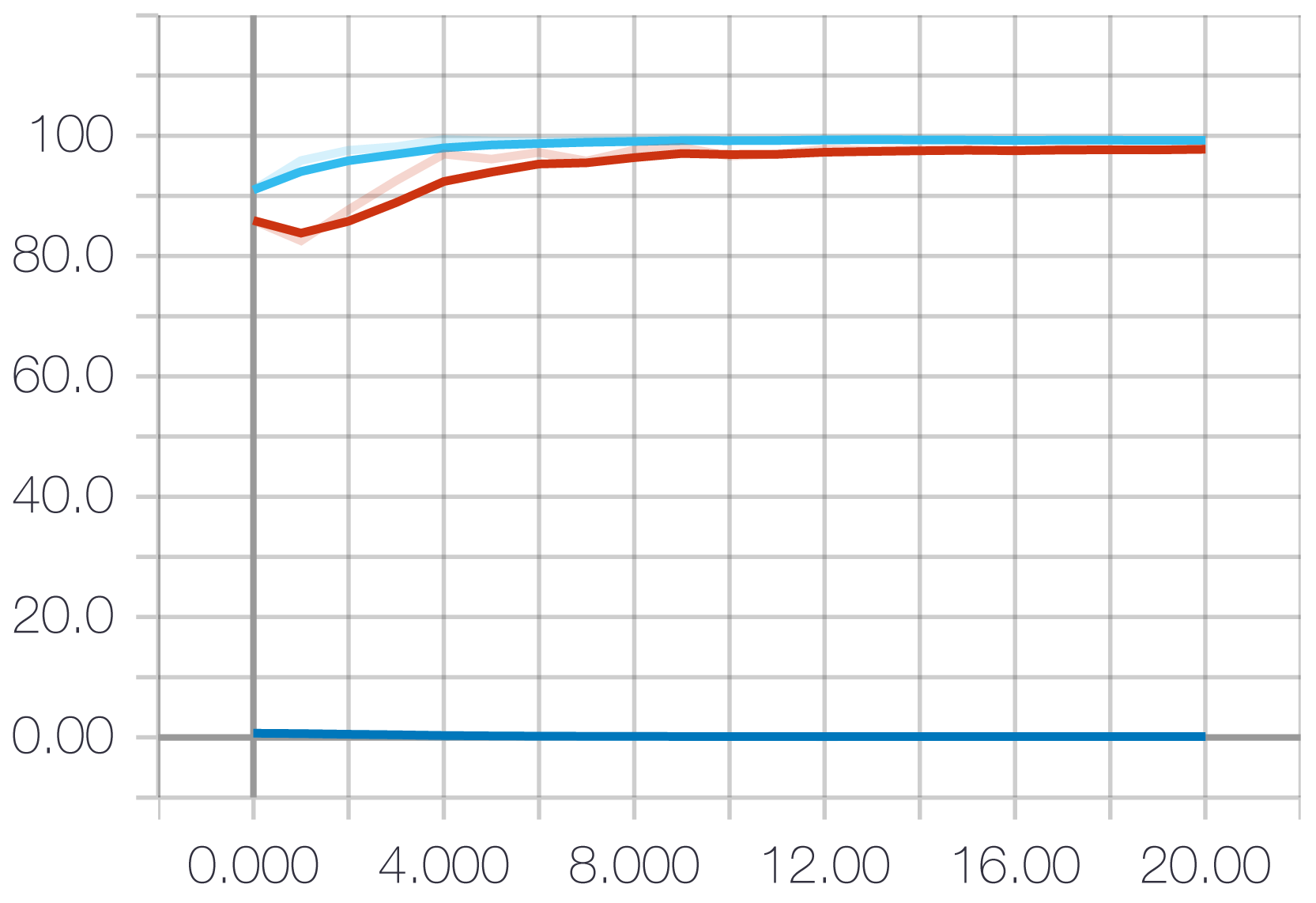

Supplement: Supplemental Information 7 [file peerj-cs-05-236-s007.zip › Source files-part3-Only for checking-To PeerJ-examiner-Please Download this Zip∩╝îAll the source files in my Manuscript-3D textures based face recognition--Author-SIMING ZHENG/6---Table-data-files---Source Document--50-101-152/data_scalar_group--101-A.tif]

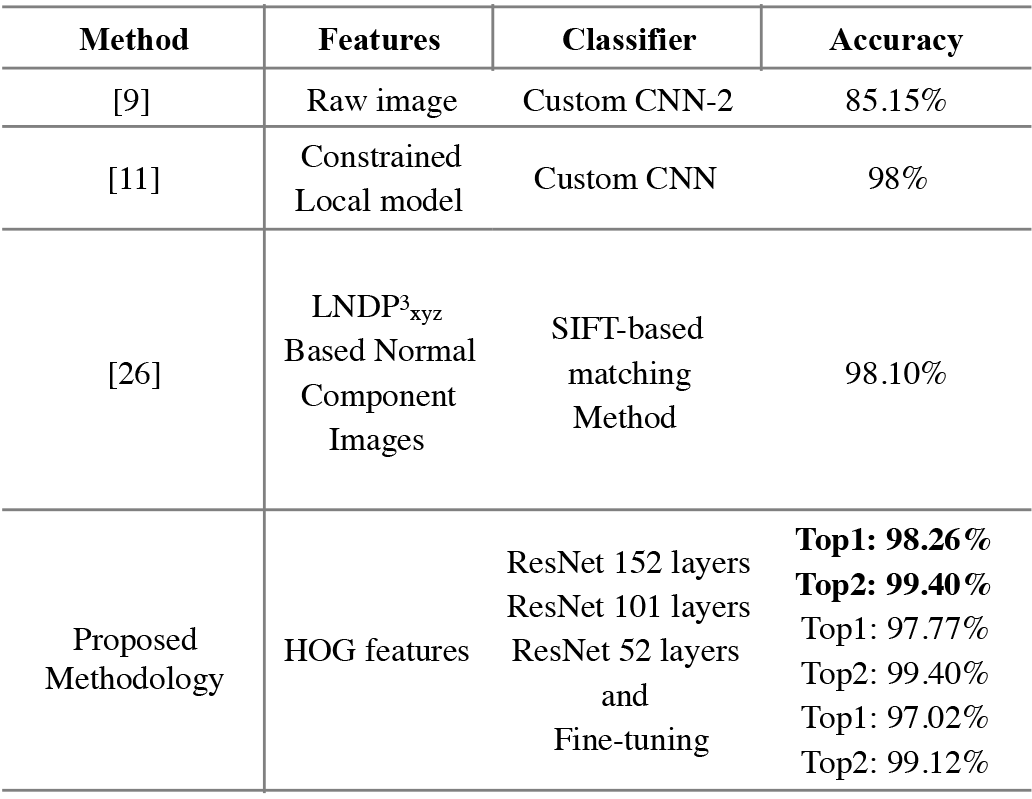

Supplement: Supplemental Information 7 [file peerj-cs-05-236-s007.zip › Source files-part3-Only for checking-To PeerJ-examiner-Please Download this Zip∩╝îAll the source files in my Manuscript-3D textures based face recognition--Author-SIMING ZHENG/6---Table-data-files---Source Document--50-101-152/compare-result.tif]

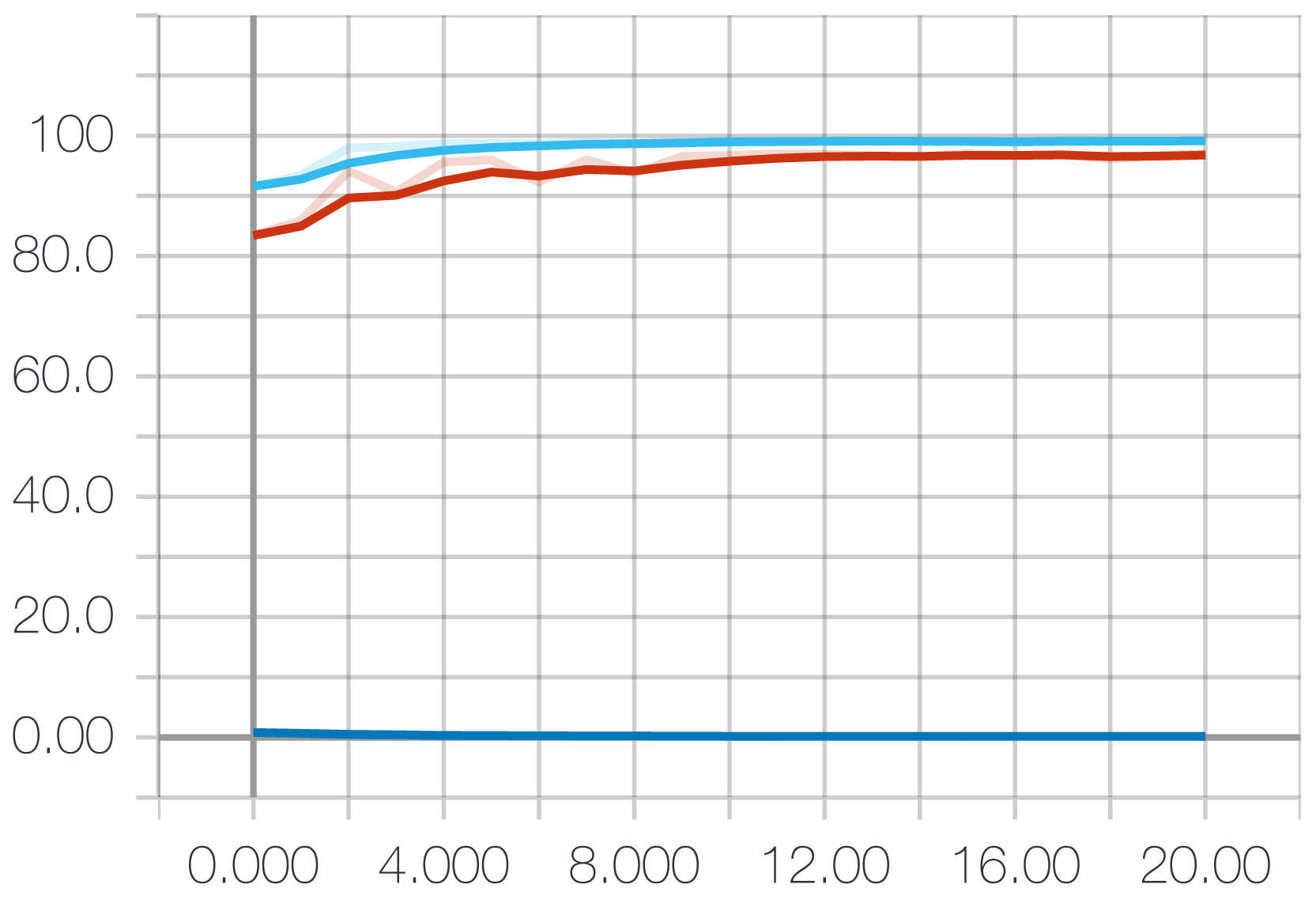

Supplement: Supplemental Information 7 [file peerj-cs-05-236-s007.zip › Source files-part3-Only for checking-To PeerJ-examiner-Please Download this Zip∩╝îAll the source files in my Manuscript-3D textures based face recognition--Author-SIMING ZHENG/6---Table-data-files---Source Document--50-101-152/data_scalar_group--50-A.tif]

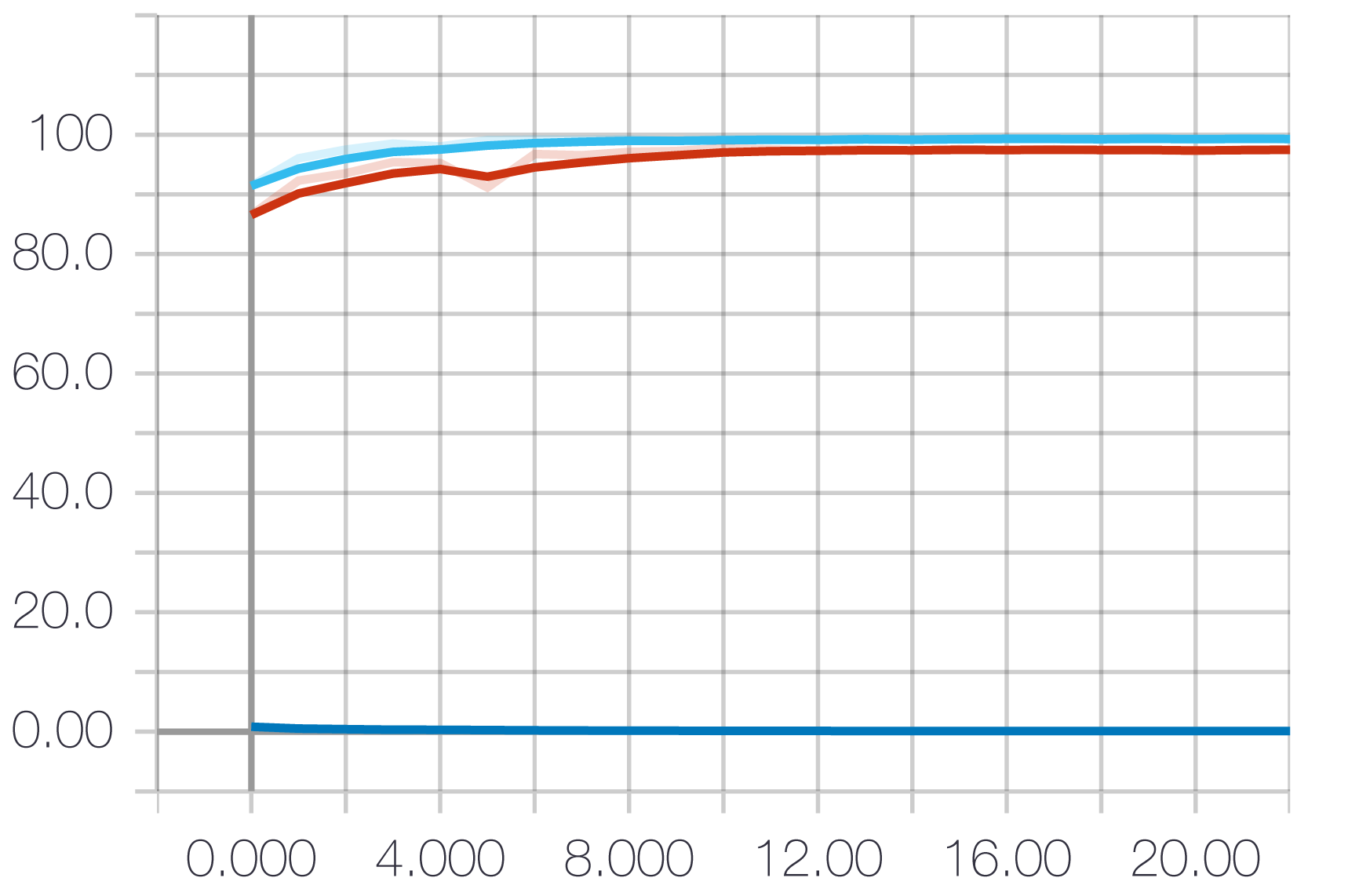

Supplement: Supplemental Information 7 [file peerj-cs-05-236-s007.zip › Source files-part3-Only for checking-To PeerJ-examiner-Please Download this Zip∩╝îAll the source files in my Manuscript-3D textures based face recognition--Author-SIMING ZHENG/6---Table-data-files---Source Document--50-101-152/data_scalar_group--152-A.tif]

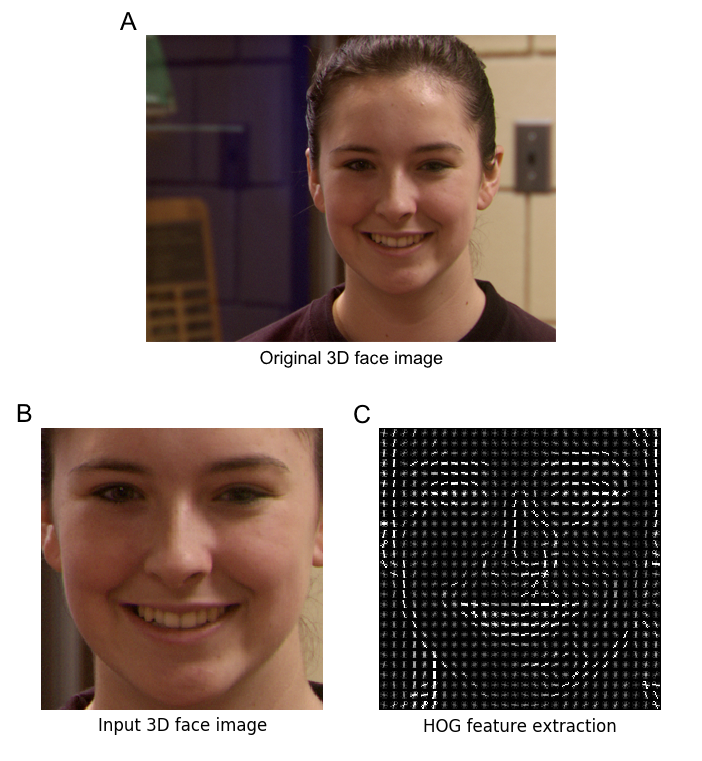

Supplement: Supplemental Information 7 [file peerj-cs-05-236-s007.zip › Source files-part3-Only for checking-To PeerJ-examiner-Please Download this Zip∩╝îAll the source files in my Manuscript-3D textures based face recognition--Author-SIMING ZHENG/5---HOG-feature-Processing-- Source Document/Fig 3. The process of HOG feature extraction..png]

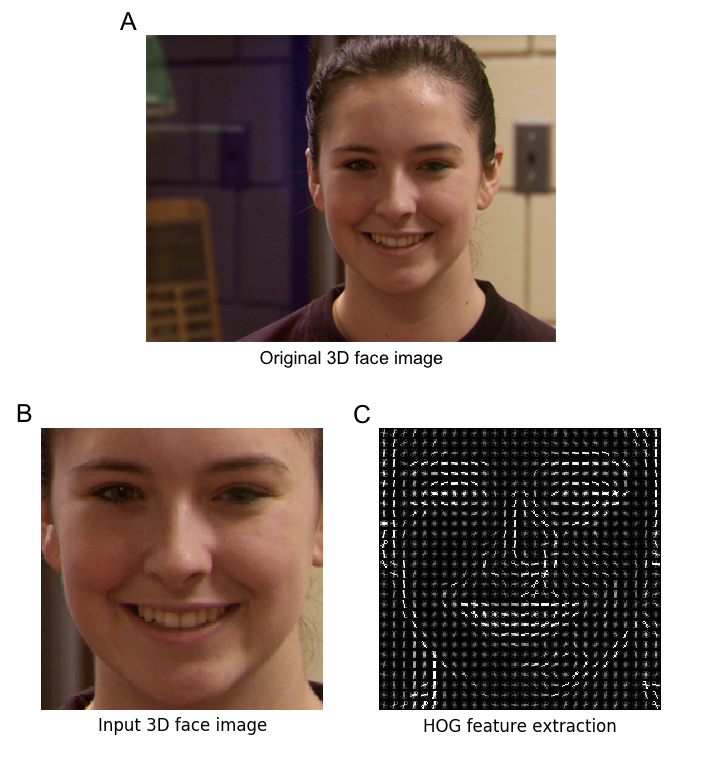

Supplement: Supplemental Information 7 [file peerj-cs-05-236-s007.zip › Source files-part3-Only for checking-To PeerJ-examiner-Please Download this Zip∩╝îAll the source files in my Manuscript-3D textures based face recognition--Author-SIMING ZHENG/5---HOG-feature-Processing-- Source Document/20 --re-re.tif]

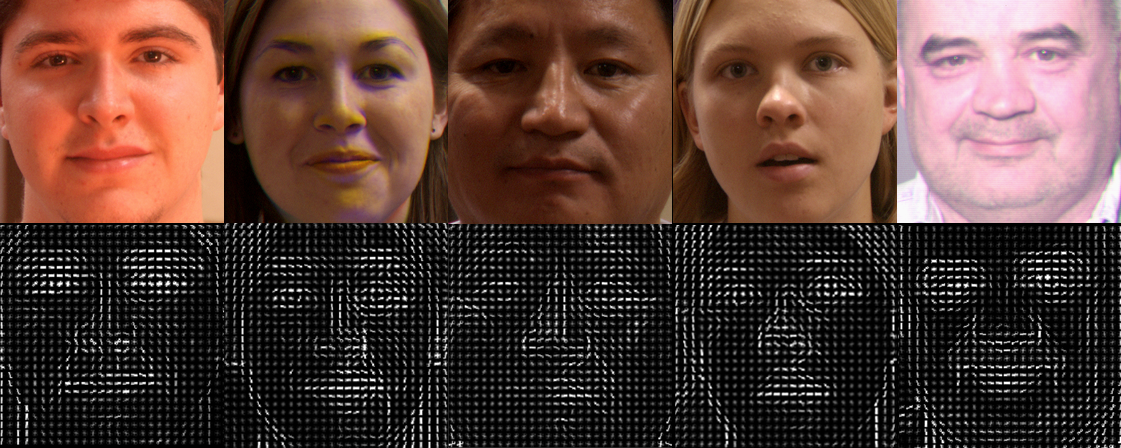

Supplement: Supplemental Information 7 [file peerj-cs-05-236-s007.zip › Source files-part3-Only for checking-To PeerJ-examiner-Please Download this Zip∩╝îAll the source files in my Manuscript-3D textures based face recognition--Author-SIMING ZHENG/5---HOG-feature-Processing-- Source Document/example-/5persons-image-HOG.tif]

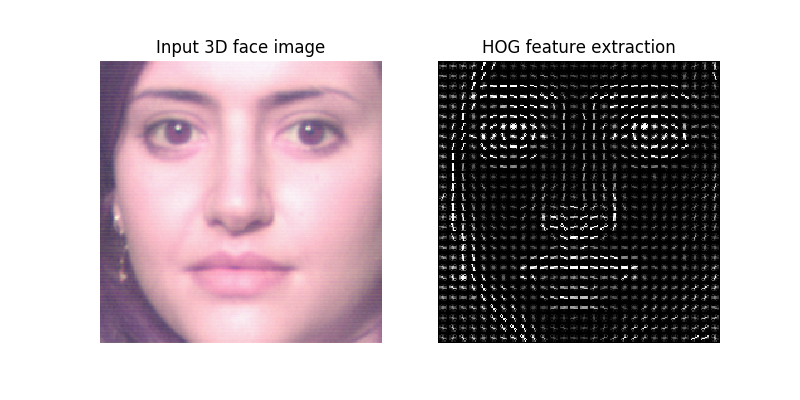

Supplement: Supplemental Information 7 [file peerj-cs-05-236-s007.zip › Source files-part3-Only for checking-To PeerJ-examiner-Please Download this Zip∩╝îAll the source files in my Manuscript-3D textures based face recognition--Author-SIMING ZHENG/5---HOG-feature-Processing-- Source Document/example-/21.png]

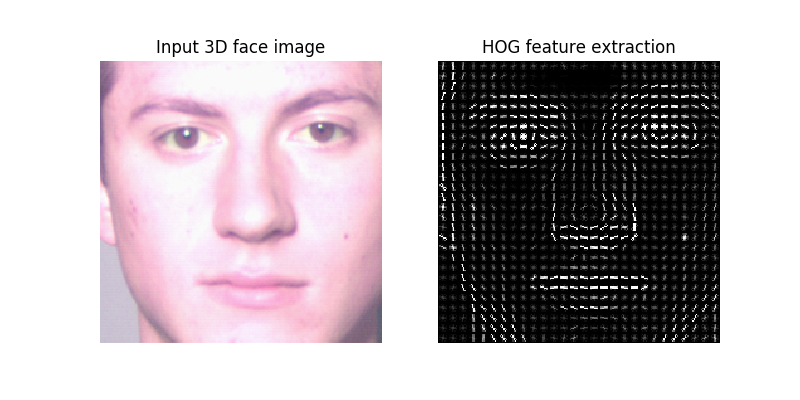

Supplement: Supplemental Information 7 [file peerj-cs-05-236-s007.zip › Source files-part3-Only for checking-To PeerJ-examiner-Please Download this Zip∩╝îAll the source files in my Manuscript-3D textures based face recognition--Author-SIMING ZHENG/5---HOG-feature-Processing-- Source Document/example-/22.png]

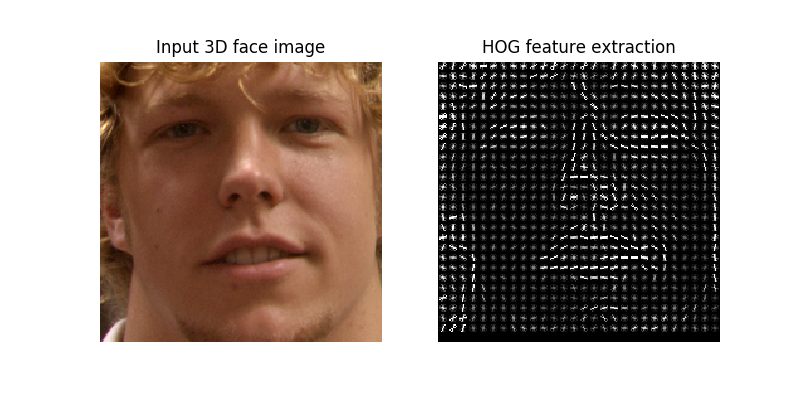

Supplement: Supplemental Information 7 [file peerj-cs-05-236-s007.zip › Source files-part3-Only for checking-To PeerJ-examiner-Please Download this Zip∩╝îAll the source files in my Manuscript-3D textures based face recognition--Author-SIMING ZHENG/5---HOG-feature-Processing-- Source Document/example-/23.png]

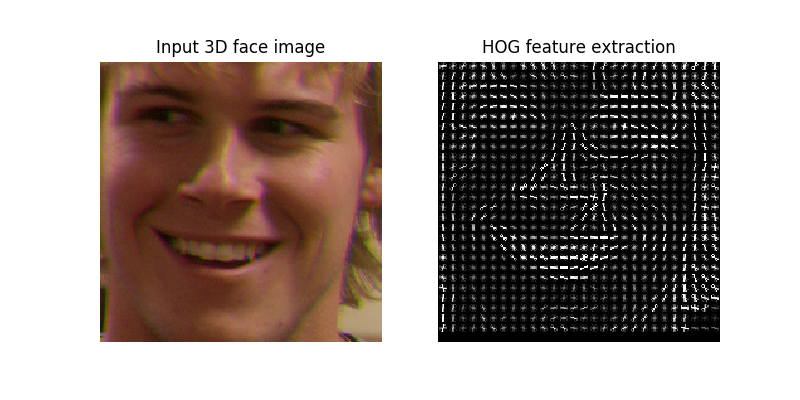

Supplement: Supplemental Information 7 [file peerj-cs-05-236-s007.zip › Source files-part3-Only for checking-To PeerJ-examiner-Please Download this Zip∩╝îAll the source files in my Manuscript-3D textures based face recognition--Author-SIMING ZHENG/5---HOG-feature-Processing-- Source Document/example-/26.png]

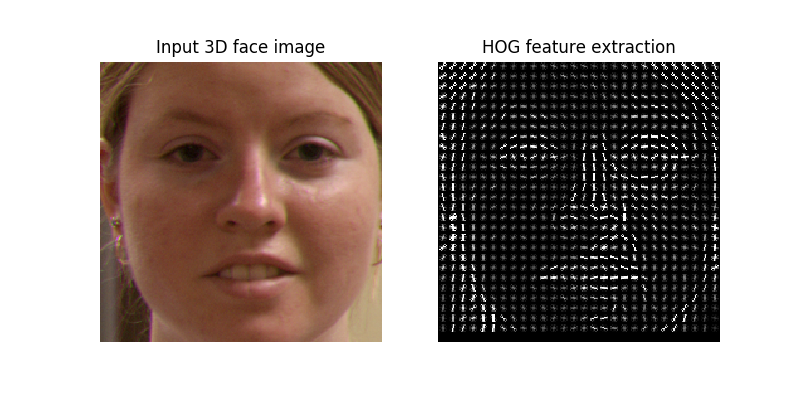

Supplement: Supplemental Information 7 [file peerj-cs-05-236-s007.zip › Source files-part3-Only for checking-To PeerJ-examiner-Please Download this Zip∩╝îAll the source files in my Manuscript-3D textures based face recognition--Author-SIMING ZHENG/5---HOG-feature-Processing-- Source Document/example-/24.png]

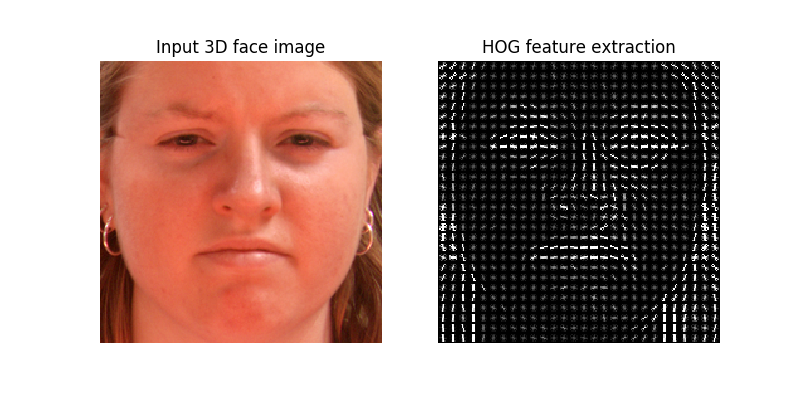

Supplement: Supplemental Information 7 [file peerj-cs-05-236-s007.zip › Source files-part3-Only for checking-To PeerJ-examiner-Please Download this Zip∩╝îAll the source files in my Manuscript-3D textures based face recognition--Author-SIMING ZHENG/5---HOG-feature-Processing-- Source Document/example-/25.png]
