# Supplementary material for: 3D texture-based face recognition system using fine-tuned deep residual networks
Source: PeerJ Comput Sci. 2019 Dec 2;5:e236. doi: 10.7717/peerj-cs.236 (PMC7924501; doi:10.7717/peerj-cs.236)
Supplement: Supplemental Information 8 [file peerj-cs-05-236-s008.docx]

----- **Explanatory Content** -----

**Literature Review and Preliminary Investigation**

The well-known 3D face database is BJUT-3D Face Database, 3DFED, BU-3DFED, GavabDB, FRGC 2.0, 3D_RMA, PRISM, CASIA-3D Face, Texas 3D Face Recognition Database, etc.

(1) BJU T-3D Face Database: It is collected by the School of Computer Science, Beijing University of Technology. The 3D face raw data was acquired using a Cyberware 3030 RGB/PS laser scanner and constructed by an alignment algorithm, including 250 men and 250 women. The database contains 3D facial information of Chinese citizens with more than 1000 neutral facial expression, including point cloud data, grid data and texture data, with high sampling accuracy.

(2) 3DFED: It is completed by Zhejiang University. A total of 40 objects of 3D face information were collected. Each object includes 3D face images of different expressions such as neutral, smiling, frowning and surprise.

(3) BU-3DFED: It is compiled by Binghamton University. The database includes 100 image acquisition objects, including 44 males and 56 females. The database is grouped by different races. The faces of the six expressions of anger, nausea, fear, happiness, sadness and surprise were collected. In addition to the neutral expression, each expression contains four levels: low, medium, high and highest. The database has 2,500 3D face images.

(4) GavabDB: It is collected by Universidad Rey Juan Carlos. The database contains 45 male and 15 female subjects, each of which collects nine three-dimensional face images, including changes in posture and expression.

(5) 3D+RMA: It is completed by SIC (Signal and Image Center). The face database contains a total of 120 acquisition objects, and each object collects six depth images. The face database is characterized by the collection of each object twice, with a time interval of 2 months. Such face data can reflect the influence of age and time factors on the 3D facial features.

(6) PRISM: Contains 166 objects. Each object's image has multiple expressions and comes from different races.

(8) CASIA-3D Face Library: This face database was collected and generated by the Institute of Automation, Chinese Academy of Sciences. The face database includes 123 face-to-face color bitmap images and 3D WRL images with different poses, expressions, and illumination changes.

(9) Texas 3D Face Recognition Database: Including 1149 2D color face images of 105 adult objects and 3D face depth images. The gender of the subject, facial expression information, and 25 manually are marked facial reference points for each face image.
